# PPAR Gamma Expression Levels during Development of Heart Failure in Patients with Coronary Artery Disease after Coronary Artery Bypass-Grafting

**DOI:** 10.1155/2014/242790

**Published:** 2014-10-13

**Authors:** Izabela Wojtkowska, Andrzej Tysarowski, Katarzyna Seliga, Janusz A. Siedlecki, Zbigniew Juraszyński, Milosz Marona, Lidia Greszata, Anna Skrobisz, Karol Kaminski, Robert Sawicki, Janina Stępińska

**Affiliations:** ^1^Intensive Cardiac Therapy Clinic, Institute of Cardiology, 42 Alpejska Street, 04-628 Warsaw, Poland; ^2^Department of Molecular Biology, Institute of Oncology, 5 Roentgena Street, 02-781 Warsaw, Poland; ^3^Department of Cardiosurgery and Transplantology, Institute of Cardiology, 42 Alpejska Street, 04-628 Warsaw, Poland; ^4^Department of Cardiology, Medical University of Bialystok, 27a Sklodowska Street, 15-276 Bialystok, Poland

## Abstract

Genetic research has elucidated molecular mechanisms of heart failure (HF). Peroxisome proliferator-activated receptors (PPARs) seem to be important in etiology of HF. The aim of study was to find the correlation between PPAR*γ* expression during development of HF in patients and coronary artery disease (CAD) after coronary artery bypass-grafting (CABG). *Methods and Results*. We followed up 157 patients (mean age 63) with CAD without clinical, laboratory, or echo parameters of HF who underwent CABG. Clinical and laboratory status were assessed before CABG and at 1, 12, and 24 months. During CABG slices of aorta (Ao) and LV were collected for genetic research. HF was defined as LVEF <40% or NT-proBNP >400 pg/mL or 6MWT <400 m. Patients were divided into 2 groups: with and without HF. PPAR*γ* expression in Ao and LV was not increased in both groups at 2-year follow-up. Sensitivity of PPAR*γ* expression in Ao above 1.1075 in detection of HF was 20.5% (AUC 0.531, 95% CI 0.442–0.619). Positive predictive value (Ppv) was 85.7%. Sensitivity and specificity of PPAR*γ* expression in the LV in detection of HF were 58% and 92.9%, respectively (AUC 0.540, 95% CI 0.452–0.626). Ppv was 73.2%. *Conclusion*. PPAR*γ* expression in Ao and LV was comparable and should not be used as predictive factor for development of HF in patients with CAD after CABG.

## 1. Introduction

According to the European Society of Cardiology (ESC) definition, heart failure (HF) is a clinical syndrome in which the patients should have the following features: symptoms typical of HF such as breathlessness or fatigue, signs of fluid retention such as pulmonary congestion or peripheral oedema, and objective evidence of a structural or functional abnormality of the heart at rest [1]. It is estimated that the overall prevalence of HF is between 2 and 3% of the European population and is steadily increasing in the recent years. There are many conditions that may lead to HF. Coronary artery disease (CAD) is by far the most common cause and is the initial precipitating condition in almost 70% of patients with HF [2, 3]. Diabetes, hypertension, high cholesterol, and smoking are the risk factors for CAD. According to data available in PubMed, 13–20% of patients develop HF after CABG procedure. An old age, female sex, diabetes, and chronic renal insufficiency are principal risk factors of HF after CABG [4–8]. Recent genetic research has attempted to elucidate molecular mechanisms of etiology and cardiac remodeling and to develop novel therapeutic strategies for heart failure. One class of molecules that are proposed to be important in the etiology of HF is the peroxisome proliferator-activated receptors (PPARs). These are ligand-activated transcription factors belonging to the nuclear hormone receptor superfamily. The PPAR superfamily is comprised of three members: PPAR*α*, PPAR*β*, and PPAR*γ*. The last one, which is the most extensively studied PPAR, has two major isoforms: PPAR*γ*1 and PPAR*γ*2, whereas at least two others, PPAR*γ*3 and PPAR*γ*4, have also been identified in multiple species including humans. It was shown that PPAR has different tissue expression. Interestingly, PPAR*γ* is expressed not only in adipose tissue but also in tissues of different origin such as coronary arteries aorta and left ventricle [9]. Regulation of PPAR receptors activity is of interest for the treatment of disorders of glucose and fatty acid metabolism. PPAR*γ* agonists are popular oral drugs for glycemic control in patients with diabetes mellitus [10]. Given that both inflammation and glucose metabolism disturbances (even those that are not considered diabetes) are risk factors of the development of HF, there is support for the notion that activity of PPARs may orchestrate the pathological changes and affect the development of HF [11].

The aim of study was to find the correlation between PPAR*γ* expression during development of HF in patients with coronary artery disease (CAD) after coronary artery bypass grafting (CABG).

## 2. Methods

We recruited and followed up patients with angiographically confirmed multivessel CAD without clinical, laboratory, and echocardiographic parameters of heart failure who underwent CABG. Patients with diabetes mellitus, prior heart failure, and valvular disease were excluded. During the surgical intervention, a small slice of the aorta and left ventricle was collected and preserved in a solution of “RNA later” (Qiagen) until further molecular analysis.

Clinical status and laboratory tests were assessed before CABG and at 1 month, 12 months, and 24 months after the surgery. Based on these results, patients were divided into two groups: group who developed HF during follow-up and those who did not. The criteria for the diagnosis of HF were left ventricle ejection fraction assessed by echocardiography <40% or NT-proBNP >400 pg/mL or six-minute walk test <400 m. None of the patients had matched these criteria prior to the surgery (Table 1).

The investigation conforms to the principles outlined in Declaration of Helsinki. The patient's informed consent and the protocol of the study were approved by the Institutional Local Ethics Committee.

### 2.1. RNA Isolation

Tissue fragments were placed in a solution of “RNA later” (Qiagen) immediately after the surgery and stored until RNA isolation. Due to the methodical difficulties of RNA isolation process (very small fragments of tissue), a RecoverAll Total Nucleic Acids Isolation kit (Ambion) was used for isolation process. The kit allowed omission of homogenization stage, during which there was significant loss of tissue material. In order to get rid of any genomic DNA remnants, the specimens were treated with DNAse. Depending on the initial amount of tissue, obtained RNA concentration varied from several dozen to several hundred nanograms. Purity ratio (A260/A280) was between 1.8 and 2.1. The cDNA was synthesized with High Capacity cDNA Reverse Transcription Kit (Applied Biosystems). For verifying the correctness of the synthesis of cDNA, the primers for the* GAPDH* gene were used. Due to small amounts of RNA and low expression level (average* ct* value 30) of investigated genes to increase the output matrix, the preamplification kit TaqMan PreAmp Master Mix (Applied Biosystems) was used. PPAR*γ* mRNA expression level was measured on the* 7500 Fast* Real-Time PCR System (Applied Biosystems) using a TaqMan method. qPCR was performed in triplicate for each gene in a 20 *μ*L reaction mix which contained 10 *μ*L of TaqMan Gene Expression Master Mix (Applied Biosystems), 1 *μ*L of each primer and probe set, 4 *μ*L of deionised water, and 5** *μ*L of diluted preamplified cDNA. The thermal cycling conditions included an initial denaturation step at 95°C for 10 min and 40 cycles of 95°C for 15 s and 60°C for 1 min. In order to avoid quantification errors associated with different amounts of template, quality, and the presence of qPCR inhibitors for accurate normalisation of real-time qPCR data, three reference genes GAPDH, TBP, and HPRT were used and geometric averaging of these genes was used for normalization. All primers and molecular probes come from Applied Biosystems which ensured the same level of amplifications efficiency and omitting DNA remnants amplification. The expression level of PPAR*γ* both in the aorta and in the left ventricle was determined in a group of 126 patients as quality of material isolated from the remaining 31 patients was too low to perform comparative analyses on both tissues sample.

### 2.2. Statistics

Data are presented as mean ± SD for quantitative variables or percent of study group for qualitative variables. Specific parameters of both groups (group with and without heart failure at baseline) and change in parameter values during follow-up were compared using chi-square test and ANOVA with post hoc analysis. Correlation between PPAR*γ* expression and other parameters was assessed with Pearson and Spearman correlation coefficients. Also all patients were divided into 2 groups according to PPAR*γ* expression value (above and below 75 percentile). The ROC curves were created to determine the cut-off point for PPAR*γ* describing patients who developed HF after CABG. A value of *P* < 0.05 was considered statistically significant. Analysis was performed using the software Statistica 10.0PL.

## 3. Results

### 3.1. Baseline Characteristics of the Patients Are Shown in Table 2

**Figure 1:**
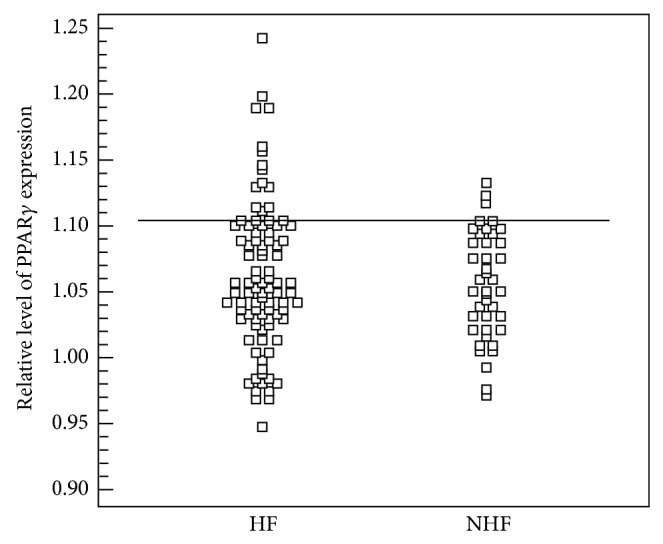
Level of expression of PPAR*γ* in aorta in the group with heart failure (HF) and without heart failure (NHF).

**Figure 2:**
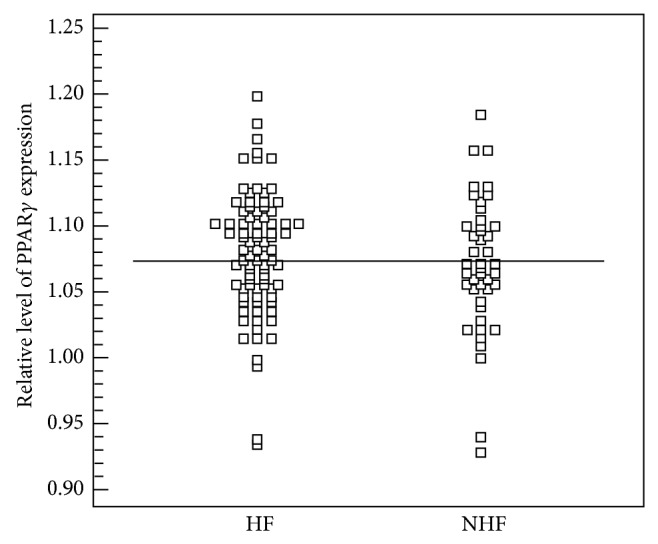
Level of expression of PPAR*γ* in left ventricle in the group with heart failure (HF) and without heart failure (NHF).

To estimate if the level of expression of PPAR*γ* predisposes patients for the development of HF, ROC curves were generated and sensitivity and specificity of the parameter were established. The level of expression of the PPAR*γ* in aorta was not very good in predicting the development of HF (AUC 0.531, 95% CI 0.442–0.619; *p* = *ns*). At the level above 1.1075, sensitivity in detecting HF was 20.5%, whereas calculated positive predictive value was 85.7%. Level of expression of the PPAR*γ* in the left ventricle sensitivity in detecting heart failure was 58% and its specificity was 92.9% (AUC 0.540, 95% CI 0.452–0.626; *p* = *ns*). Established positive predictive value was 73.2% (Figures 1 and 2).

**Figure 3:**
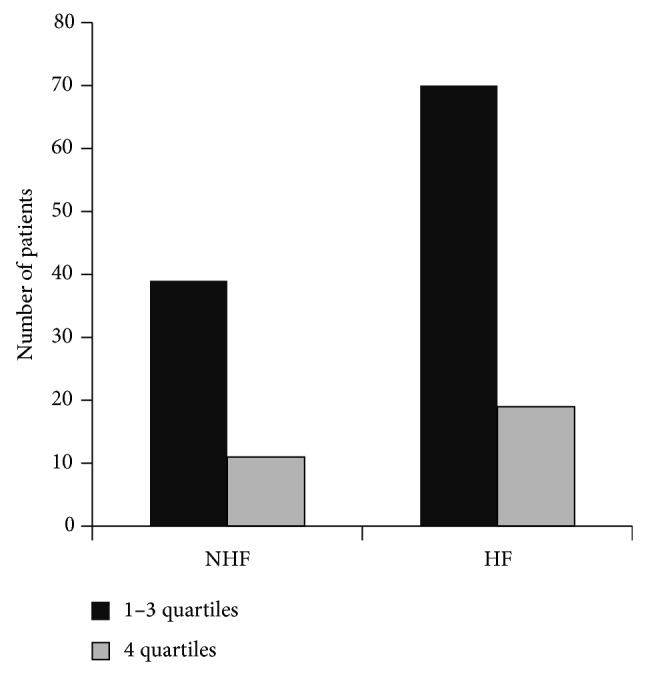
Number of patients within the 4th quartile against the other three quartiles in the group with (HF) and without heart failure (NHF). HF: heart failure, NHF: without heart failure, LV: left ventricle, and A: aorta.

All patients have been divided into two groups: those with and without HF. The temporal changes of crucial clinical parameters in patients with HF and without heart failure (NHF) are presented in Table 1. In both groups, we have analysed the frequency of patients within the 4th quartile of PPAR*γ* expression against the other three quartiles, but there were no significant differences (*P* < 0.896) (Figure 3).

### 3.2. Study Limitation

There were some study limitations. First, 15 patients withdrew informed consent and further 41 patients did not return for follow-up evaluation. Second, due to ethical issues, we were unable to collect the tissue samples in 2-year follow-up. These additional data could determine long-term changes of level of expression of PPAR*γ* and provide valuable information regarding pathogenesis of HF.

## 4. Discussion

The role of PPAR*γ* in the development of HF is complex and controversial. Although there are numerous studies available, still little is known about its role in development of HF in humans. Most of the studies have been conducted on animals or in vivo models. Recent clinical studies raise the question of action of PPAR*γ* synthetic ligands (thiazolidinedione, prostaglandins) on the cardiovascular system. These synthetic factors may interfere with and influence expression of PPAR*γ*. On one hand, PPAR*γ* agonists reduce atherosclerosis in human patients and animal models [12–15]. A large body of preclinical studies indicates that, in addition to their effect on atherogenesis, PPAR*γ* ligands also impact on CAD and the development of HF. Prolonged ischemia leads to cardiomyocyte death which is followed by a series of structural and functional alterations in the viable myocardium, known as cardiac remodeling. Adaptive changes in the extracellular matrix and in cardiomyocyte biology occur, which are initially able to maintain contractile function. However, progressive cardiac remodeling leads to chamber dilatation, contractile dysfunction, and ultimately heart failure [16]. Human genetic studies on PPAR*γ* have revealed that functional changes in this nuclear receptor are associated with CAD. Moreover, PPAR*γ* ligands reduce the hypertrophy caused by mechanical strain in neonatal cardiac myocytes [17]. On the other hand, there is clinical evidence of an increase in the incidence of HF in patients with type 2 diabetes mellitus that are treated with thiazolinediones (TZDs) [18] and mortality following myocardial infarction in rats [19].

Son et al. showed that increased level of PPAR*γ* expression in transgenic mice caused hypertrophy and dysfunction of the left ventricle due to increased lipotoxicity [20]. There were no such abnormalities observed in transgenic mice with low expression of PPAR*γ*. Chintalgattu et al. demonstrated in their study with cardiac fibroblasts that PPAR*γ* agonists induce expression of VEGF which plays an important role in ischaemic myocardium. These findings suggest that the PPAR*γ* agonists may have positive impact on cardiac remodelling [21]. There are also other clinical evidences that PPAR*γ* agonists improve contractility and diastolic function of the left ventricle [22, 23].

Most of evidences for an important role of PPAR*γ* in development of HF come from studies in animal models. In contrast, we present large prospective study with 157 participating patients. We have demonstrated that 28 (17%) of patients developed HF over a period of two years. The progression of atherosclerosis, leading to acute ischaemic episodes (in 71%) and AF (in 29%), was the main cause of HF. For further genetic examination, tissue samples were obtained directly from aorta and left ventricle during CABG. We demonstrated the expression of PPAR*γ* both in aorta and in left ventricle. However, we did not confirm results from previous study, which showed increased level of expression of PPAR*γ* in the left ventricle and age-related receptor expression in aorta in human donor hearts [24]. It could be explained by the small population (five patients) of the previous study. Surprisingly, our results did not show increased level of expression of PPAR*γ* neither in group in which it came to the development of HF nor in the group without subsequent HF (mean ± 1.065, mean ± 1.054 versus mean ± 1.054, mean ± 1.076) in two-year follow-up.

At the level of expression of the PPAR*γ* in aorta above 1.1075, sensitivity in detecting HF was more than 20.5%, while calculated positive predictive value was 85.7%. Level of expression of the PPAR*γ* in the left ventricle sensitivity in detecting heart failure was 58% and its specificity was 92.9%. Established positive predictive value was 73.2%. These values disqualify the analysis of PPAR*γ* tissue expression as prognostic factor. Given the marked discrepancy with previously published experimental data, this study provides first so extensive evidence in humans that PPAR*γ* expression in tissue does not predict the development of HF.

## 5. Conclusions

Our findings imply that PPAR*γ* expression in aorta and LV was comparable and should not be used as predictive factor for development of HF in patients with CAD treated with CABG.

## Figures and Tables

**Table 1:** Temporal changes of crucial clinical parameters in patients with (HF) and without heart failure (NHF).

	HF				NHF			
	1 month *n* = 93	1 year *n* = 66	2 years *n* = 62	*P*	1 month *n* = 46	1 year *n* = 64	2 years *n* = 39	*P*
NT-proBNP ng/mL (±SD)	647.6 (±410.2)	414.8 (±401.5)	423.0 (±453.5)	0.0001	221.9 (±97.9)	205.1 (±215.7)	236.4 (±238.0)	0.25
6MWT m (±SD)	369.7 (±99.7)	465.9 (±107.9)	438.7 (±128.1)	0.00002	444.9 (±53.3)	500.2 (±77.9)	489.5 (±107.8)	0.054
LVEF < 40%, *n* (%)	12 (14%)	16 (24%)	14 (18%)	0.03	0 (0%)	2 (3%)	0 (0%)	0.9
PPAR gamma Ao, (±SD)	1.065 (±0.053)			NS	1.054 (±0.077)			NS
PPAR gamma LV, (±SD)	1.079 (±0.051)			NS	1.076 (±0.051)			NS

**Table 2:** Baseline patients characteristics.

Parameters	*n* = 157
Mean age, yrs (±SD)	63.8 (±8.81)
Sex/men, *n* (%)	133 (85%)
BMI, kg/m^2^ (±SD)	27.1 (±3.10)
Prior MI, *n* (%)	56 (36%)
Anterior wall	13 (8%)
Inferior wall	32 (21%)
Posterior wall	8 (5%)
Lateral wall	6 (4%)
Hypertension, *n* (%)	98 (62%)
Hypercholesterolaemia, *n* (%)	157 (100%)
Killip class, *n* (%)	
I	157 (100%)
HR, min^−1^ (±SD)	68.7 (±7.64)
BPsys, mmHg (±SD)	131.8 (±12.20)
BPdias, mmHg (±SD)	79.1 (±5.15)
Creatinine, umol/L (±SD)	89.0 (±31.9)
MDRD, mL/min/1.73 m^2^ (±SD)	74.2 (±14.7)
Hemoglobin, g/dL (±SD)	13.56 (±1.98)
Hematocrit, % (±SD)	39.4 (±6.90)
NT-pro BNP, ng/m (±SD)	195.0 (±101.98)
6MWT, m (±SD)	443.0 (±58.51)
EF < 40%, *n* (%)	0
LV > 5.6 cm, *n* (%)	0
ASA, *n* (%)	155 (99%)
B-blockers, *n* (%)	154 (99%)
ACE-I, *n* (%)	144 (92%)
ARB, *n* (%)	4 (3%)
Diuretics, *n* (%)	20 (13%)
Inhibitors of aldosterone, *n* (%)	10 (6%)
Statins, *n* (%)	153 (98%)
